# Highly Polygenic Variation in Environmental Perception Determines Dauer Larvae Formation in Growing Populations of *Caenorhabditis elegans*

**DOI:** 10.1371/journal.pone.0112830

**Published:** 2014-11-13

**Authors:** James W. M. Green, Jana J. Stastna, Helen E. Orbidans, Simon C. Harvey

**Affiliations:** Biomolecular Research Group, School of Human and Life Sciences, Canterbury Christ Church University, Canterbury, United Kingdom; Centre National de la Recherche Scientique & University of Nice Sophia-Antipolis, France

## Abstract

**Background:**

Determining how complex traits are genetically controlled is a requirement if we are to predict how they evolve and how they might respond to selection. This requires understanding how distinct, and often more simple, life history traits interact and change in response to environmental conditions. In order to begin addressing such issues, we have been analyzing the formation of the developmentally arrested dauer larvae of *Caenorhabditis elegans* under different conditions.

**Results:**

We find that 18 of 22 previously identified quantitative trait loci (QTLs) affecting dauer larvae formation in growing populations, assayed by determining the number of dauer larvae present at food patch exhaustion, can be recovered under various environmental conditions. We also show that food patch size affects both the ability to detect QTLs and estimates of effect size, and demonstrate that an allele of *nath-10* affects dauer larvae formation in growing populations. To investigate the component traits that affect dauer larvae formation in growing populations we map, using the same introgression lines, QTLs that affect dauer larvae formation in response to defined amounts of pheromone. This identifies 36 QTLs, again demonstrating the highly polygenic nature of the genetic variation underlying dauer larvae formation.

**Conclusions:**

These data indicate that QTLs affecting the number of dauer larvae at food exhaustion in growing populations of *C. elegans* are highly reproducible, and that nearly all can be explained by variation affecting dauer larvae formation in response to defined amounts of pheromone. This suggests that most variation in dauer larvae formation in growing populations is a consequence of variation in the perception of the food and pheromone environment (*i.e.* chemosensory variation) and in the integration of these cues.

## Background

Complex traits are a consequence of variation at multiple contributing loci that each individually explain only a fraction of trait variance [1]. Hence, for many such traits, the additive effect of a contributing locus is often so small that quantitative trait loci (QTL) or association studies lack the statistical power to identify them [2]. Other traits are determined by a relatively small number of loci - including a large number of human diseases. Whilst QTLs and the underlying quantitative trait nucleotides (QTNs) that contribute to them can be detected [3]–[5], in most cases these are of relatively large effect. Such loci are therefore essentially Mendelian in nature. It is therefore unclear to what extent current approaches are identifying ecologically and evolutionarily relevant variation [6]–[8]. How environmental variation acts on genotype-to-phenotype maps further complicates such questions, particularly given issues concerning the difficulty of identifying any adaptive benefits of such genotype by environment interactions [9], [10].

Environmental responses, formally examples of phenotypic plasticity, can be either continuous across a given range of conditions or discontinuous, appearing as switches between distinct phenotypes. Conceptually, polyphenic forms of phenotypic plasticity can be thought of as conditional strategies [11], with organisms comparing environmental cues to genetically specified thresholds [12], [13]. Understanding how phenotypic plasticity evolves and how it is maintained by selection is required if we are to understand life-history evolution [14]. It is therefore necessary to understand the underlying genetic pathways that control a given polyphenism and how that specific pathway is affected by other variation in other traits. Recently, there has been much progress in the analysis of the mechanisms controlling developmental switches in plants (e.g. [15], [16]). In general, there is however either a good understanding of the genetics or of the ecology of such models, but not both.

A case in point is dauer larvae development in the nematode *Caenorhabditis elegans*. This striking developmental switch, found within many nematodes, involves a worm either developing directly to adulthood or arresting development as a dauer larva, an alternate third larval stage [17]. Dauer larvae are stress-resistant, long-lived, and appear to be central to dispersal between food patches in *C. elegans* [18]–[20]. The genetic pathways that control dauer larvae development are well characterized (for review see [21]) and the propensity, or not, of developing worms to enter the dauer larval stage has been used as a model of phenotypic plasticity (e.g. [22], [23]) and of signaling in intraspecific interactions [24]. Similar analyses have also been undertaken in *Pristionchus pacificus* [25], [26], but in both species, understanding of the ecology is comparatively limited.

For the appropriate induction of dauer larvae development, worms must accurately predict what the environment will be when they complete development. This involves the integration of information about the number of worms in the environment, estimated based on the amount and composition of dauer pheromone present, and the availability of food [21], [27]. Both pheromone and food are assessed by the direct perception of chemicals from the environment. In the wild, dauer formation will occur in a dynamic environment where the food availability and levels of pheromone will change due to worm population growth, pheromone dispersal and degradation, the growth of bacterial food, and the actions of other species. Comparisons between wild isolates of *C. elegans* have identified extensive variation between wild isolates in dauer larvae formation in response to defined amounts of pheromone [22], [24], assessed by determining the proportion of worms from an age matched cohort that develop as dauer larvae under defined food and pheromone conditions. Variation between wild isolates has also been observed in dauer larvae formation in growing populations [28], as assessed by determining the number of dauer larvae present at the time of exhaustion of a fixed food source. For both of these assay types, the underlying genetics has been investigated, with analysis of dauer larvae formation in response to defined amounts of pheromone in recombinant inbred lines (RILs) produced from crosses between DR1350 and N2 identifying 3 QTLs [23]. Analysis of the number of dauer larvae at food exhaustion in growing populations of introgression lines (ILs) with regions of the CB4856 genome introgressed into an N2 background revealed a more complex picture, identifying 24 QTLs affecting dauer larvae development [28]. Given these results, it is not clear if this contrast in QTL numbers is a consequence of the differences in mapping methodologies (ILs vs RILs), reflects differences between the parental isolates (CB4856 vs DR1350), or indicates that more traits affect the number of dauer larvae at food exhaustion in growing populations. As dauer larvae development is important both for understanding the ecology of *C. elegans* and as a model for phenotypic plasticity, we sought to address this issue. We first retested previously identified QTLs affecting the number of dauer larvae at food exhaustion in growing populations (here referred to as QTLs gp1-24) and investigated their effects in additional environmental conditions. These assays show that these QTLs are reproducible and that the food patch size affects the ability to detect one class of QTLs. We then analyzed, using the same ILs, dauer larvae formation in response to defined amounts of pheromone. This identifies 36 QTLs, again demonstrating the highly polygenic nature of the genetic variation underlying dauer larvae formation. Comparison of the QTLs identified in these two screens indicates that most QTLs affecting the number of dauer larvae at food exhaustion in growing populations overlap with QTLs affecting dauer larvae formation in response to defined amounts of pheromone. We also show that a laboratory derived allele of *nath-10* affects the number of dauer larvae at food exhaustion in growing populations, but not dauer larvae formation in response to defined amounts of pheromone.

## Results

### Food patch size affects the ability to detect QTLs and estimates of effect size

**Figure 1:**
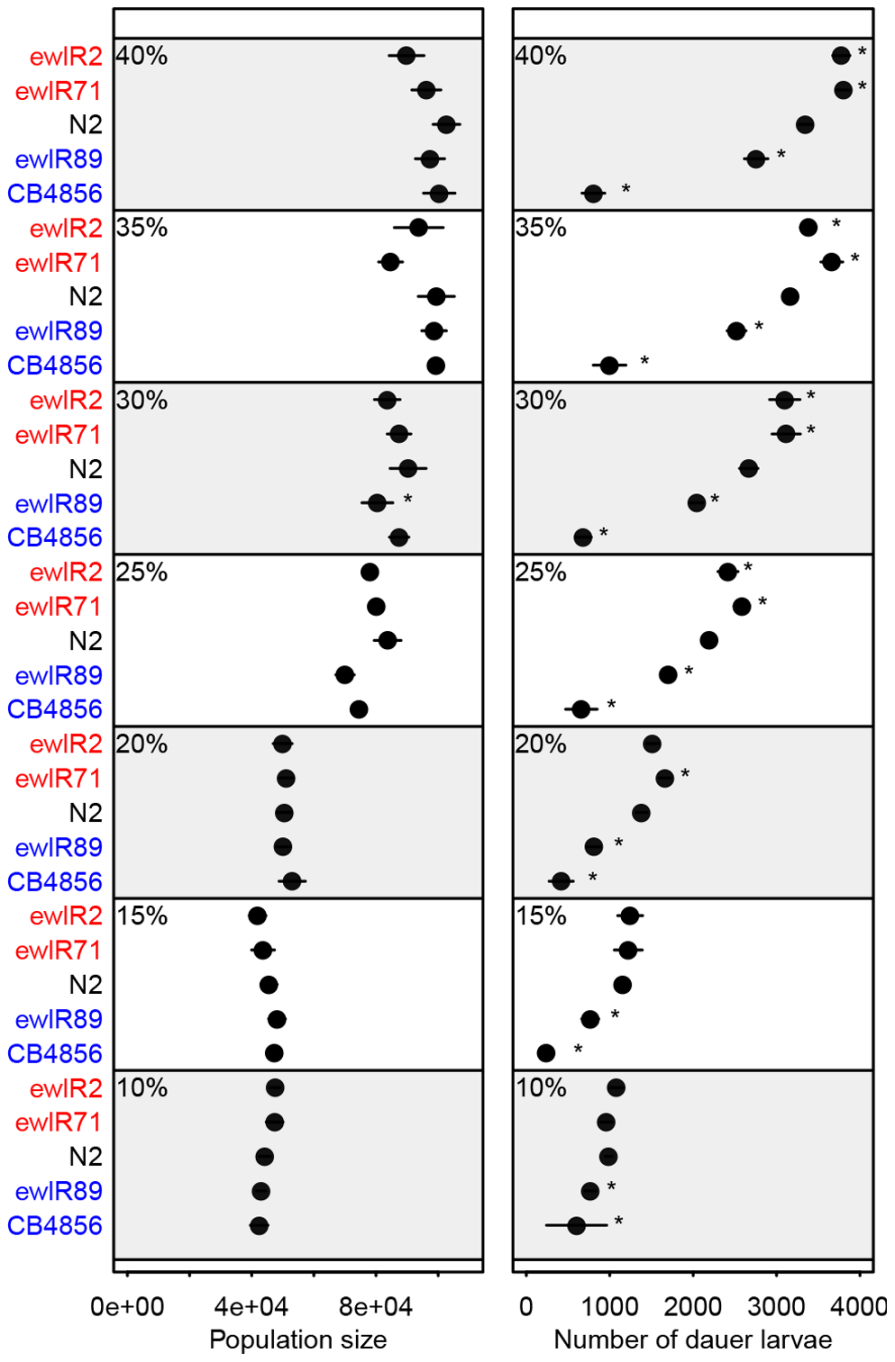
Food patch size affects the ability to detect QTLs and estimates of effect size. The mean population size (A) and number of dauer larvae (B) at exhaustion of 100 µl of a range of % w/v food patches for N2, ILs containing positive (ewlR2 and 71) and negative (ewlR89) effect QTLs and CB4856. Error bars indicate ±1 S.E. and asterisks indicate significant difference to N2 at that food concentration (*p*<0.05, *post hoc* testing by Fisher’s Least Significant Difference for population size and Mann-Whitney U-test for the number of dauer larvae).

The number of dauer larvae at food exhaustion was determined for two groups of ILs containing previously identified dauer larvae formation QTLs (see [28]). Analyses of these data indicates that QTLs that result in fewer dauer larvae than N2, negative effect QTLs, are highly reproducible, whilst those that result in more dauer larvae than N2, positive effect QTLs, are not (Table 1). Note that block effects, principally a consequence of differences between batches of *E. coli*, make direct comparisons between specific food concentrations in different assays difficult, hence comparisons are always made to control lines within an assay. Similarly, these differences between assays make drawing conclusions about the absolute effect of QTLs on dauer larvae numbers difficult and hence our analysis focuses on the direction of the effect. To explore why positive effect QTLs are difficult to replicate, comparisons were made of a subset of the ILs at a range of food concentrations (Figure 1). These data indicate that variation between genotypes in population size at each food concentration is limited and that the food patch size influences the ability to detect QTLs affecting the number of dauer larvae at food exhaustion (Figure 1). Specifically, ewIR89, which contains a negative effect QTL, can be distinguished from N2 at all food concentrations tested. In contrast, the two ILs containing positive effect QTLs (ewIR2 and ewIR71) are detected consistently only when there are higher numbers of dauer larvae. This indicates that QTLs resulting in more dauer larvae than N2 are reproducible, but only within a subset of environments.

**Table 1:** Reproducibility of QTLs affecting dauer larvae formation in growing populations.

Chromosome	QTL	QTLEffect	Line(s)	20%w/v^1^	20%w/v1^1^	20% w/v	20% w/v	Samesign	1 week afterexhaustion	Support
***I***	gp1	P	2			781	**−672**	N	**2491**	Y
	gp2	N	6				**−1083**	Y		
	gp3	P	13vs14						**−**544	N
	gp4	N	13			**−**1036	**−688**	Y	**−**636	Y
***II***	gp5	N	19				**−912**	Y		
	gp7	N	26			**−3614**	**−571**	Y	**−2066**	Y
	gp8	P	24			**−**1019	**−538**	N	**1603**	Y
	gp9	P	26vs27						**2680**	Y
***III***	gp10	P	40vs36						**−**1502	N
	gp11	N	40				**−757**	Y	2222	N
	gp12	P	40vs41						251	N
***IV***	gp13	N	45			**−3132**		Y		
		N	46				**−**466			
		N	48			**−3725**	**−1947**			
	gp14	N	53			**−**358	**−376**	Y		
	gp16	N	58				**−**366	Y		
		N	59			**−1939**	**−456**			
***V***	gp17	P	69				**−**363	N		(see Fig. 1)
		P	71				**−434**			
		P	72			**−**570	**−584**		827	
	gp18	N	76			**−2792**	**−422**	Y		
***X***	gp19	N	78	**−353**	**−**442	**−**925		Y		Y
		N	79	**−445**	**−1087**		**−539**		**−1483**	
		N	81	**−624**	**−1194**		**−525**			
	gp20	P	79vs80						**1870**	Y
	gp21	N	81	**−624**	**−1194**		**−525**	Y		
	gp22	N	81	**−624**	**−1194**		**−525**	Y		
		N	83	**−455**	**−882**	**−**119				
		N	85	**−612**	**−1178**	**−3386**	**−**198			
		N	86	**−403**	**−**156					
	gp23	P	87	**326**	**−**200	1344	**−574**	N	**1587**	Y
	gp24	N	89	**−556**	**−356**	**−3303**		Y		
		N	90	**−317**	**−478**					

**Figure 2:**
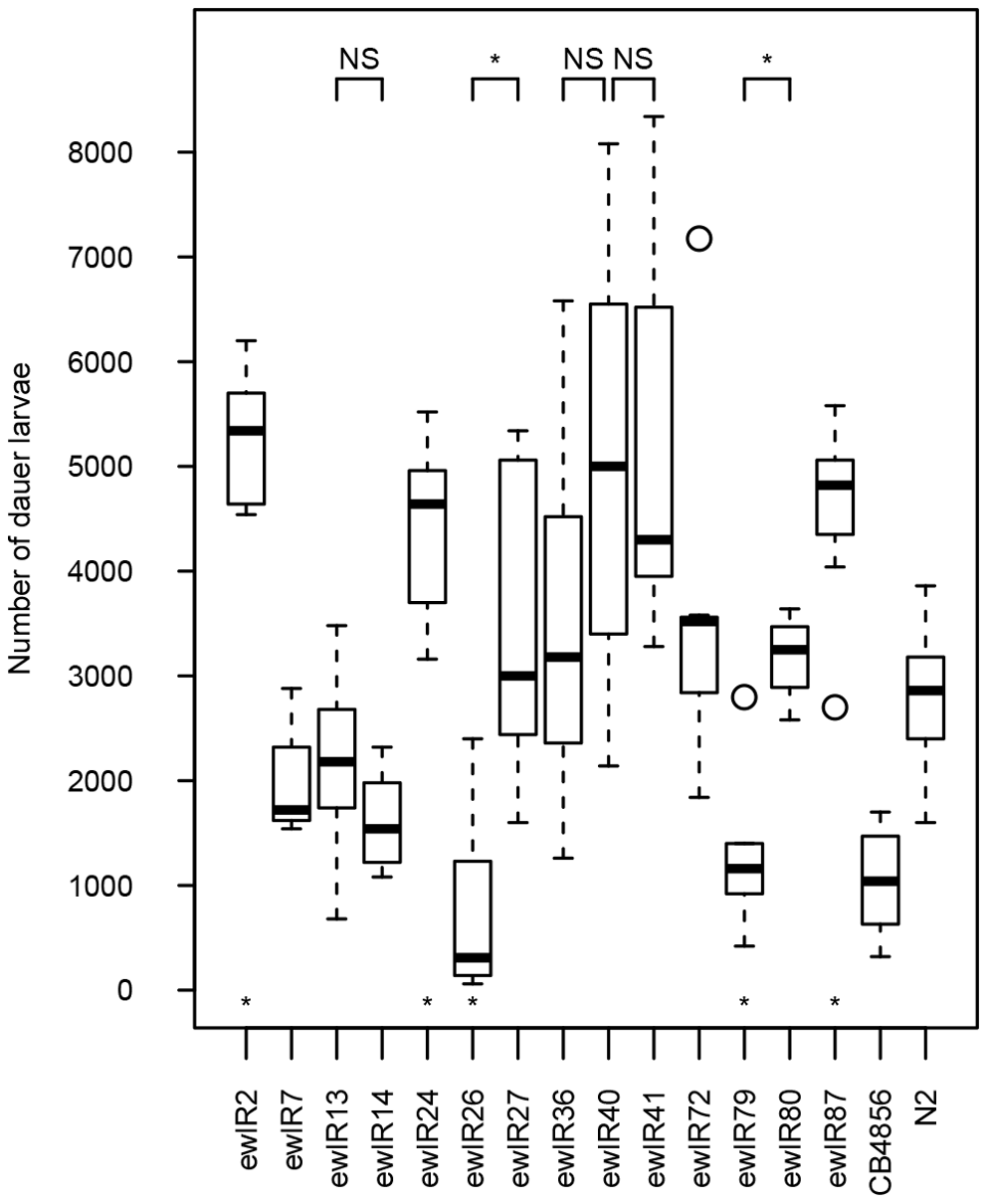
Introgression lines vary in the number of dauer larvae present a week after food exhaustion. Box and whisker plots of the number of dauer larvae present a week after exhaustion of 100 µl of 20% w/v food in N2, CB4856 and in ILs containing both positive and negative effect QTLs. CB4856 is shown only as a reference. ILs that differed from N2 are indicated by asterisks above the line names (*p<*0.05, *post hoc* Mann-Whitney U-tests). IL versus IL comparisons are shown at the top of the figure, with asterisks denoting significant differences between ILs tested (*p*<0.05, Mann-Whitney U-tests with a Bonferroni adjustment to correct for multiple testing), comparisons where the ILs do not differ are labelled NS.

Shown is the difference in dauer larvae number between the IL and N2 from that assay, or between two ILs, for two re-tests of ILs with introgressions on the X chromosome for the number of dauer larvae at food exhaustion (data from [28]), two assays for the number of dauer larvae at food exhaustion, and an assay of dauer larvae numbers a week after food exhaustion (see Figure 2). Values in bold are significantly different (*post hoc* comparison by Mann-Whitney U-test, *p*<0.05) in the relevant comparison (N2 vs IL or IL vs IL). ‘QTL effect’ indicates if the initial genome-wide screen identified the QTL as positive or negative, ‘Same sign’ shows if the difference is in the same direction in all tests at food exhaustion. ‘Support’ indicates in the assay of dauer larvae numbers a week after food exhaustion identified a QTL with the same effect as the original screen. Note that QTLs gp1 and 17 are detected in Figure 1.

To further investigate this, we determined the number of dauer larvae present a week after food exhaustion, a situation known to increase the number of dauer larvae in N2 [29]. In this assay, analysis of N2 and CB4856 at food exhaustion indicated that only very few dauer larvae had formed at this point (mean ± SE of 12.5±3.66 in N2, with no dauer larvae observed in CB4856, n = 8 for both). As expected, a week after food exhaustion many more dauer larvae had developed (Figure 2). Analysis of these data indicates that the number of dauer larvae varies by genotype (Figure 2: H = 59.37, df = 14, *p*<0.001) and shows that many of the positive effect QTLs identified by Green et al [28] can be detected under these conditions (Table 1).

In combination, these analyses retest 22 of the 24 QTLs previously identified [28] and provide support for 18 of them (Table 1). Of the 4 QTLs not supported in these assays (gp3, 10, 11 and 12), 3 are a consequence of the different behavior of ewlR40 in the assay for the number of dauer larvae present a week after food exhaustion (Table 1). As gp11, the QTL identified in ewlR40, is supported by one of the screens for the number of dauer larvae at food exhaustion (Table 1), it is possible that this difference reflects a biological distinction between assays rather than the QTL not being present, *i.e.* ewlR40 behaves differently to other assayed ILs after food exhaustion.

### Variation at *nath-10* affects the number of dauer larvae at food exhaustion in growing populations

**Figure 3:**
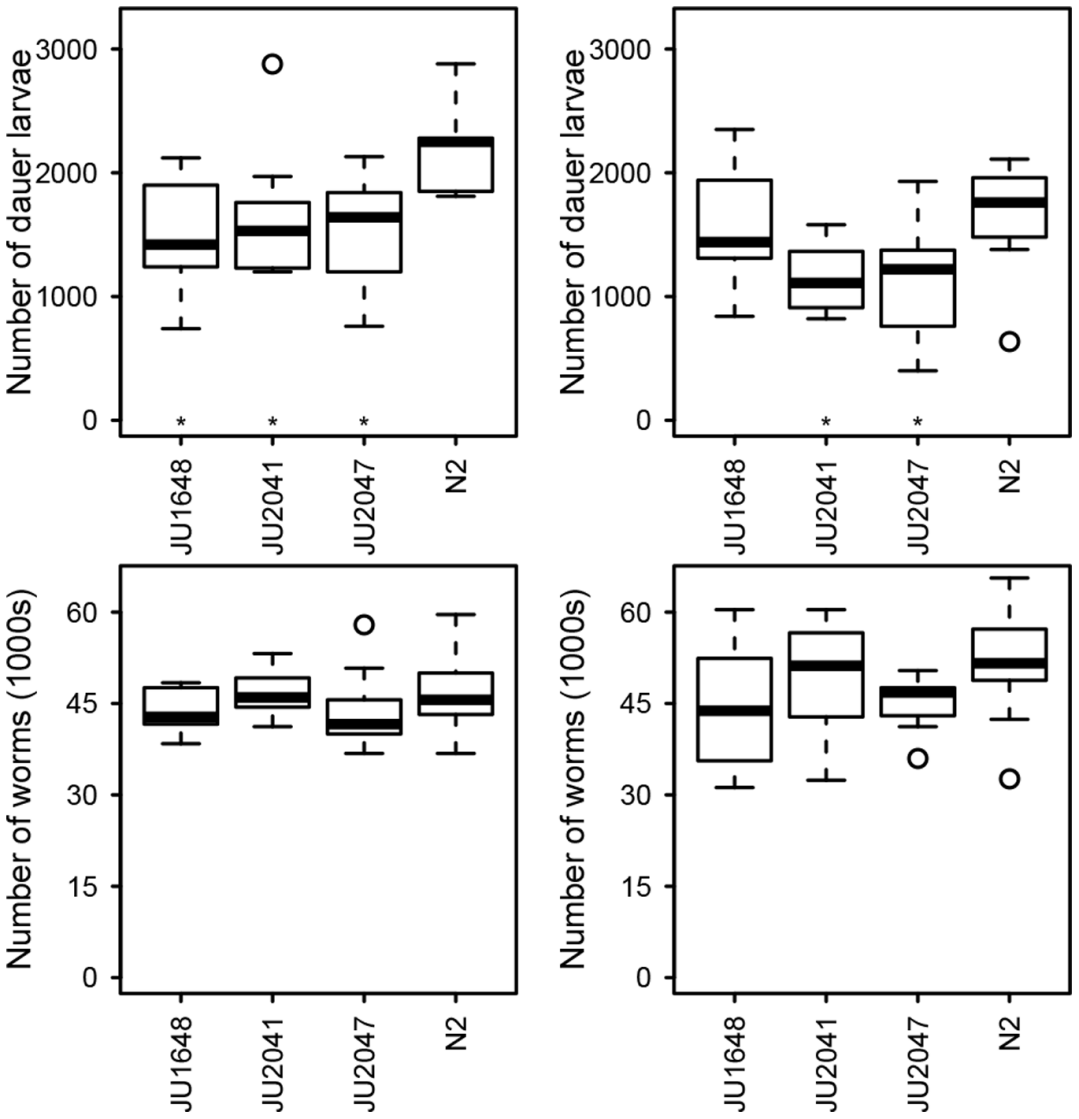
Variation surrounding *nath-10* affects the number of dauer larvae at food exhaustion in growing populations. Box and whisker plots showing number of dauer larvae (top panels) and the population size (bottom panels) at exhaustion of 100 µl of 20% w/v food in two assays (left and right columns) in N2 and in JU1648, JU2041 and JU2047, lines with very fine introgressions of the *nath-10(haw6805)* allele in an N2 genetic background. The *nath-10(haw6805*) allele is the only common difference that distinguishes JU2041, JU2047, and JU1648 from N2, and JU2041 contains only two known alleles that differ from N2, *nath-10(haw6805)* and *mfP19* (see [19] for further details). Asterisks indicate significant difference to N2 within that assay (*p*<0.05, *post hoc* testing by Fisher’s Least Significant Difference).

Given that a laboratory derived allele of *npr-1* affects dauer larvae development in growing populations [28], we investigated if other alleles that have arisen during the time N2 has spent in culture in the laboratory (see Table S3 in [30]) could affect this trait. Comparison of the QTL limits to candidate laboratory alleles with non-synonymous amino acid substitutions (see Table S3 in [30]) revealed candidate genes for QTLs gp2 (*nath-10*, identified in [30] as F55A12.8, and *gld-2*), gp10 (K10D2.1, K04G7.1 and F56C9.11), gp15 (*pept-1,* identified in [30] as *opt-1*) and gp21 (C46C11.4 and F39C12.1, with this QTL also containing *npr-1*). As variation at *nath-10* has been shown to affect age at maturity, brood size, and egg-laying speed [31], traits that might be expected to affect the properties of growing populations, this polymorphism was further investigated. No evidence was identified to link the polymorphisms in other genes to either reproductive or dauer development traits, so these were not further investigated here. Analysis of data from lines that vary at *nath-10* indicated that population size at food exhaustion was lower in lines carrying the *nath-10(haw6805)* allele, but did not differ between isolates in either assay (Figure 3: F_3,32_ = 0.85, *p* = 0.48, F_3,31_ = 1.27, *p* = 0.30 for assays 1 and 2, respectively). In contrast, the number of dauer larvae at food exhaustion differed between lines in both assays (Figure 3: F_3,32_ = 4.96, p = 0.006, F_3,31_ = 3.64, *p* = 0.02 for assays 1 and 2, respectively), with more dauer larvae observed in N2 than all other lines in assay 1 and more than JU2041 and JU2047 in assay 2 (Fisher’s LSD). This indicates that variation in the region of *nath-10* affects dauer larvae formation in growing populations. As the *nath-10(haw6805)* allele is the only common difference distinguishing JU2041, JU2047, and JU1648 from N2, and JU2041 contains only two known alleles that differ from N2, *nath-10(haw6805)* and *mfP19* [19], the most parsimonious explanation is that the differences observed here are a consequence of variation at *nath-10*.

### Introgression lines differ in dauer larvae formation in response to defined amounts of pheromone

**Figure 4:**
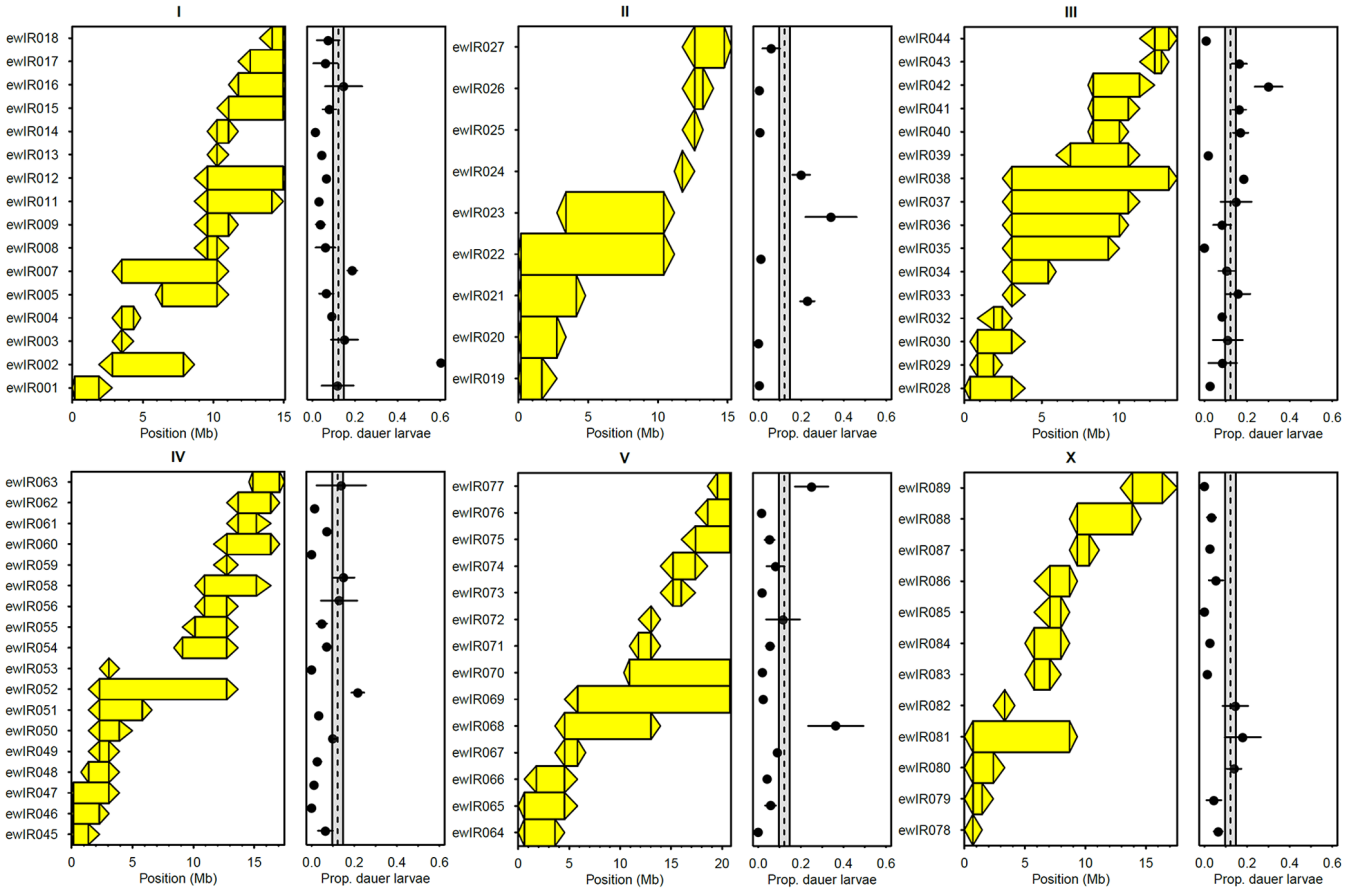
Introgression lines differ in dauer larvae formation in response to defined amounts of pheromone. Shown for each chromosome are the ILs assayed (left hand panels), with the CB4856 introgression per IL shown by the colored rectangle and triangles joining adjacent CB4856 and N2 markers, and the proportion of dauer larvae that developed in each IL (right hand panels) in dauer larvae formation assays with 25 µl of dauer pheromone extract and 20 µl of 1% w/v *E. coli*. Error bar represent ±1 S.E. and the dashed lines and shaded bars represent the mean ±1 S.E. of the N2 controls.

Analysis of dauer larvae formation in standard dauer larvae assays [22], [23], [27], where an age-matched cohort of larvae develop in the presence of defined amounts of food and pheromone, indicates that fewer worms develop as dauer larvae in many of the ILs than in N2 (Figure 4). This identifies a large number of QTLs, with 42 of the 76 ILs assayed differing from N2 (Figure 4, Table S1). Comparison between ILs indicated that dauer larvae formation differed between ILs in 32 of the 56 tested pairs of ILs (Table S2). In combination, these analyses identify a total of 36 QTLs affecting dauer larvae development in response to defined amounts of pheromone (Table 2; Table S3). Of these, 17 QTLs result in higher dauer larvae with the CB4856 allele (positive effect) and 19 result in a lower dauer larvae formation with the CB4856 allele (negative effect).

**Table 2:** Locations and effect of QTLs detected for dauer larvae formation in response to defined amounts of pheromone.

Chromosome	QTL	Comparison(s)	QTLeffect	QTL limits(Mbp)	Overlap
I	1	2	P	1.8–3.5	gp1
	2	7, 5vs7, 4vs7	P	4.3–6.4	
	3	14v9	P	8.7–10.3	
	4	9, 13, 14	N	9.6–11.1	gp4
	5	17v16	P	11.1–12.6	
	6	17	N	11.8–15.1	
II	7	22, 23vs22	N	0–3.4	gp5
	8	21, 23	P	2.8–4.8	gp6
	9	21v22	N	4.1–11.2	
	10	25, 26	N	11.8–13.2	gp7
	11	24	P	11.2–12.6	gp8
	12	26v27	P	13.2–15.3	gp9
III	13	28, 30v28	N	0–0.84	
	14	39v37	P	2.5–6.8	gp10
	15	35, 40v36, 33v35,39, 41v39	N	5.9–8.3	gp11
	16	42, 35v36	P	9.3–10.6	gp12
	17	41v42	P	10.6–12.3	gp12
	18	44, 43v44	N	12.7–13.8	
IV	19	46, 47, 49v48, 48,52, 45v46, 50	N	1.4–2.3	gp13
	20	49v50	N	3.1–5.0	gp14
	21	51, 50v51	P	3.9–6.6	
	22	51v52, 55v54, 54	N	8.4–10.1	gp15
	23	59v56	P	10.1–12.7	
	24	58, 60, 56v58, 62,59v60, 61v62	N	15.2–16.4	(gp16)
	25	61	P	12.7–16.4	
V	26	66, 69	N	4.6–5.8	
	27	68, 71v68, 67v68	P	5.8–11.8	
	28	70, 72v71, 71	N	10.9–13.0	
	29	73, 77v76	N	14.0–17.4	gp18
	30	76	N	17.4–20.9	
	31	77	P	18.6–20.9	
X	32	86v81, 79v80	P	1.5–3.3	gp20
	33	83, 85	N	5.8–8.0	gp22
	34	85v86	P	8.0–9.3	gp23
	35	87	N	8.7–11.1	
	36	88, 89	N	12.9–14.6	gp24

‘QTL effect’ indicates if the CB4856 genotype at the QTL increases (P) or decreases (N) the number of dauer larvae in comparison to N2. ‘QTL Limits’ denote the maximum possible QTL region defined by these comparisons. ‘Overlap’ denotes QTLs that colocalize with QTLs affecting dauer larvae development in growing populations.

**Figure 5:**
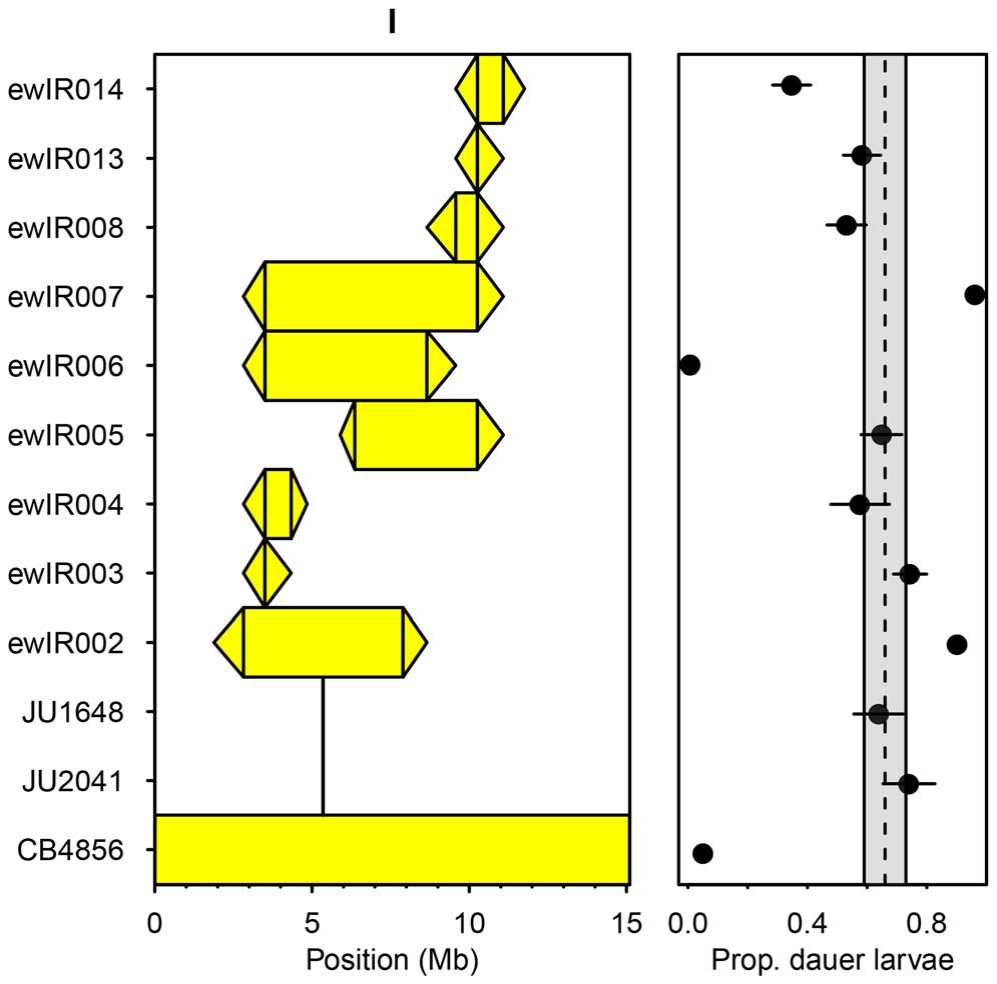
Variation at *nath-10* does not affect dauer larvae formation in response to defined amounts of pheromone. Reanalysis of ILs with introgressions in the region of *nath-10* and of JU1648 and JU2041, lines with very fine introgressions of *nath-10(haw6805)*. The *nath-10(haw6805)* allele is the only common difference distinguishing JU2041 and JU1648 from N2, and JU2041 contains only two known alleles that differ from N2, *nath-10(haw6805)* and *mfP19* [19]. The CB4856 introgression per IL is shown by the colored rectangle and triangles joining adjacent CB4856 and N2 markers (left hand panel), and the proportion of dauer larvae that developed in each line (right hand panel) in dauer larvae formation assays with 40 µl of dauer pheromone extract and 20 µl of 1% w/v *E. coli*. Error bars represent ±1 S.E. and the dashed lines and shaded bar represent the mean ±1 S.E. of N2.

Reanalysis of ILs with introgressions on chromosome I and of JU1648 and JU2041, lines with very fine introgressions of *nath-10(haw6805)* (see above and the Methods for line details), indicates that polymorphism at *nath-10* does not affect dauer larvae formation in response to defined amounts of pheromone (Figure 5: comparison of N2, JU1648 and JU2041, F_2,20_ = 0.67, *p* = 0.53). The ILs are however variable (Figure 5: comparison of N2 and the ILs, F_9,61_ = 29.93, *p*<0.001), with ewlR2 and ewlR7 showing more dauer larvae than N2 and ewlR6 and ewlR14 showing fewer dauer larvae than N2 (Fisher’s LSD). This recovers the QTLs detected in the genome-wide screen (Figure 4, Table 2, Table S4) and also indicates that there are additional negative effect alleles within the introgression in ewlR6, as the reduced dauer larvae formation in this line cannot be a consequence of variation at *nath-10*.

**Figure 6:**
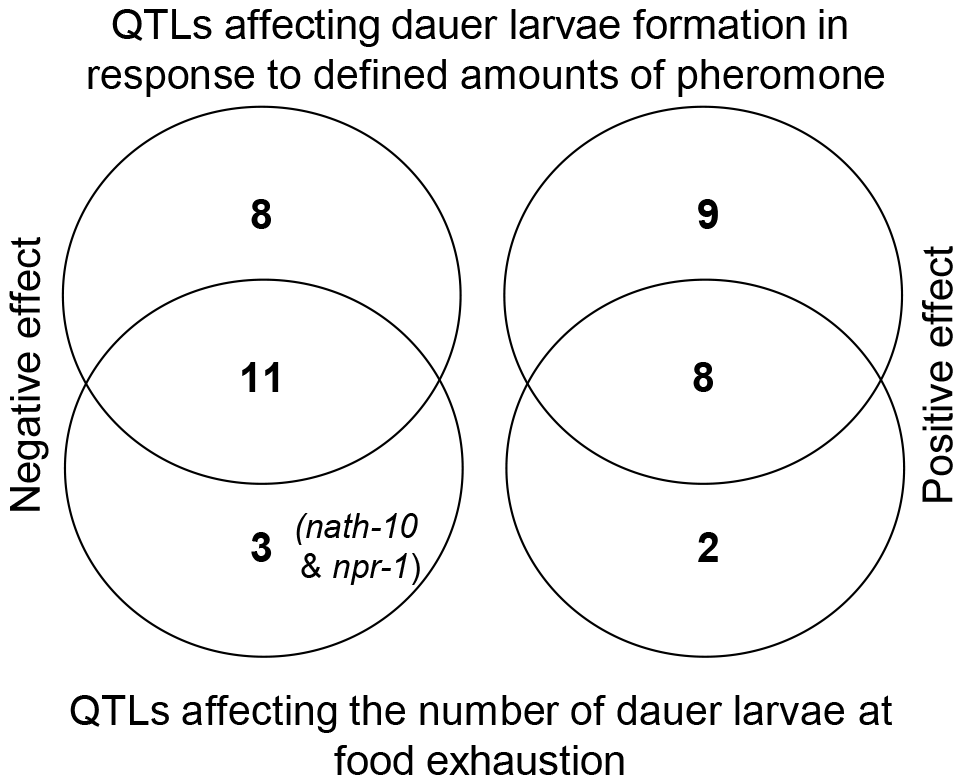
Most QTLs affecting the number of dauer larvae at food exhaustion in growing populations overlap with QTLs affecting dauer larvae formation in response to defined amounts of pheromone. Venn diagrams showing the overlap between QTLs that affect dauer larvae development in response to defined amounts of pheromone (upper circles) and QTLs that affect the number of dauer larvae at food exhaustion in growing populations (lower circles). Negative effect QTLs, where the CB4856 allele decreases the number of dauer larvae, are shown in the left hand diagram and positive effect QTLs, where the CB4856 allele increases the number of dauer larvae, are shown in the right hand diagram.

Comparison of the QTLs detected in growing populations (Table 1, [28]) with those affecting dauer larvae formation in response to defined amounts of pheromone (Figure 3, Table 2) indicates that the majority of QTLs affecting the number of dauer larvae in growing populations co-localize with those that affect dauer larvae formation in response to defined amounts of pheromone (Table 2, Figure 6). Of the QTLs that only affect the number of dauer larvae at food exhaustion in growing populations, gp3 is not supported in this analysis (Table 1), and another, gp21, is at least in part due to variation at *npr-1* [28], a polymorphism that does not affect the likelihood of dauer larvae formation in standard dauer assays [22].

## Discussion

These data indicate that QTLs affecting dauer larvae formation in growing populations of *C. elegans* are highly reproducible. Analysis of ILs containing previously identified QTLs [28] reveals that negative effect QTLs, which reduce the number of dauer larvae at food exhaustion in growing populations, are robust and can be recovered under a wide range of environmental conditions (Table 1; Figure 1). In contrast, the positive effect QTLs are more difficult to replicate and are only apparent in a subset of environments (Table 1; Figure 1), with analysis at different levels of food indicating that they are only consistently revealed at higher concentrations of food. Analysis of dauer larvae formation at increased levels of food availability also more clearly differentiates between CB4856 and the ILs containing negative effect QTLs, *i.e.* many negative effect QTLs phenocopy CB4856 at low food concentrations (Figure 1, also see [28]), but are different to CB4856 at higher food concentrations (Figure 1). Many of these QTLs also affect dauer larvae formation after food exhaustion (Figure 2).

That the QTLs affecting dauer larvae in growing populations are reproducible does not however tell us how the differences between ILs are produced. In theory, differences in dauer larvae formation in growing populations could result from differences in the reproductive traits that determine population size and hence affect pheromone production and food consumption. Alternatively, differences could result from variation in traits that determine dauer development by affecting the perception of the food and pheromone environment and the integration of this information. Analysis of dauer larvae formation in response to defined amounts of pheromone identifies a total of 36 QTLs (Figure 4; Table 2), and these QTLs are sufficient to explain the majority of the growing population QTLs (Figure 6). Given the nature of these dauer larvae formation assays, these QTLs cannot be a consequence of differences in traits that affect population growth rates. They are also unlikely to be a consequence of traits that affect either pheromone production or food consumption as there is limited opportunity for such variation to affect the levels of food and pheromone in these assays.

Given the number of chemoreceptors in *C. elegans* and the diversity between isolates [32] it is a strong possibility that chemoreceptor diversity underlies many of these QTLs. For instance, many annotated chemoreceptor pseudogenes in N2 have only one apparent defect, and appear functional in a least some wild isolates [32]. There are also extensive larger scale differences between isolates of *C. elegans*. For example, array comparative genomic hybridization indicates that 517 genes present in N2 are deleted, or sufficiently divergent that they cannot be detected, in CB4856 [33], [34]. We therefore expect that many of the QTLs we detect are a consequence of variation in chemoreceptors.

That the majority of the growing population QTLs can be explained by variation in dauer larvae formation in response to defined amounts of pheromone (Figure 6) implies that variation in reproductive traits is not the primary cause of variation in dauer larvae formation in growing populations. As many reproductive parameters are variable between CB4856 and N2, and between the ILs used here (e.g. [35]–[38]), this suggests that there may be constraints that prevent reproductive traits from affecting the development of dauer larvae in growing populations. For instance, population growth may be highly correlated with both food consumption and with pheromone production, meaning that the changes in the population growth rates do not alter the relative levels of food and pheromone at the critical points in population development.

There are however QTLs that we only detect in one of the screens (Figure 6). As we detect more QTLs affecting dauer larvae formation in response to defined amounts of pheromone than QTLs affecting the number of dauer larvae at food exhaustion, we consider it likely that we have underestimated the number of QTLs affecting dauer larvae formation in growing populations. *i.e.* we would expect that some of the QTLs affecting dauer larvae formation in response to defined amounts of pheromone do affect dauer larvae formation in growing populations, but were just not detected previously. It is also possible that some are environment-specific and are a consequence of the differences between the two types of assay (*e.g.* growing population assays were done at 20°C whilst the dauer larvae formation assays were done at 25°C). The five QTLs that do affect dauer larvae formation in growing populations, but do not affect dauer larvae formation in response to defined amounts of pheromone (Table 2; Figures 5 and 6), might also be a result of environment-specific effects. Two of these are however, at least in part, the result of identified polymorphisms in *nath-10* (Figures 3 and 5) and *npr-1* [28]. Another of the QTLs that are only detected in the growing population assays, gp3, is not reproduced in this work (Table 2; Figure 2). There is therefore little evidence for environmental specificity for these QTLs.

That variation at *nath-10* does not affect dauer larvae formation in response to defined amounts of pheromone agrees with previous work that found no effect of the *nath-10(haw6805)* allele on high-temperature induced dauer larvae formation [31]. This indicates that one of the other effects of variation at this locus must underlie the effects seen on dauer larvae numbers at food exhaustion in growing populations. It is however not clear why the variation at *nath-10*, where the N2 allele increases sperm number and egg laying rate [31], affects dauer larvae formation in growing populations independently of population size (Figure 3). Given that dauer larvae formation in growing populations does not appear to be closely related to variation in population size [28], this may suggest that variation at *nath-10* has additional effects on the worms. Alternatively, the reproductive effects of variation at *nath-10* may impact of the population dynamics in ways that are not captured by this assay.

In combination, analysis of *C. elegans* dauer larvae formation in two distinct assays identifies many QTLs ([28] and this study), indicating that the control of this trait is highly polygenic. In comparison to other studies that have looked at the genetics of natural variation in *C. elegans* (e.g. [3], [23], [31], [37]–[42]) this suggests both that mapping in ILs allows the identification of more QTLs and also that the control of variation in dauer larvae formation is affected by more varying genes than many other assayed traits. Modeling predicts that traits under moderate selection, rather than either weak or strong selection, are more likely to be encoded by many loci with highly variable effects [43]. The large number of QTLs observed here may therefore indicate that dauer larvae formation has been under this type of selection.

In the wild, large growing populations of *C. elegans* can be found on rotting fruits and herbaceous stems [19]. Whilst these wild populations are generally smaller (up to 10′s of thousands of individuals) than those observed here (see Figure 1), they do contain large numbers of dauer larvae and pre-dauer second stage larvae [19]. This implies that dauer larvae formation in growing populations in the wild is more likely than in such populations in laboratory conditions. As conditions that result in increased dauer larvae formation (high food and assessment after food exhaustion, Table 1 and Figures 1 and 2) increase the observed effect of the positive effect QTLs we detect and allow us to distinguish between CB4856 and ILs containing negative effect QTLs, we hypothesise that the QTLs we detect here would be likely to affect dauer larvae formation in the wild. These data do however highlight the fact that much variation between isolates of *C. elegans* is only apparent under more stressful conditions than are normally experienced in the laboratory.

## Conclusions

Complex traits are a consequence of variation in many distinct component life history traits. Here we demonstrate that the control of dauer larvae formation in *C. elegans* is highly polygenic, that QTLs affecting dauer larvae formation in growing populations are highly reproducible, and that most can be explained by variation affecting dauer larvae formation in response to defined amounts of pheromone. This indicates variation at the level of the growing population is principally a consequence of variation in just one class of underlying response, *i.e.* in traits that affect the perception of the food and pheromone environment and the integration of these cues rather than variation in reproductive traits that determine population size. We also show that variation at *nath-10* affects dauer larvae formation in growing populations.

## Methods

### Worm lines

N2 was obtained from the *Caenorhabditis* Genetics Centre. JU1648, JU2041 and JU2047 [31], isolates with introgressions of the *nath-10(haw6805)* allele in an N2 genetic background, were obtained from Marie-Anne Félix (IBENS). The *nath-10(haw6805)* allele is the only polymorphism that is common to JU2041, JU2047, and JU1648, and that distinguishes these lines from N2 [19]. JU2041 contains only two known alleles that differ from N2, *nath-10(haw6805)* and the LSJ1 allele of *mfP19*, with the LSJ1 allele of *mfP19* not found in either JU2047 or JU1648 [19]. The construction of the CB4856/N2 ILs is described in [44] and these lines were obtained from Jan Kammenga (Wageningen University). Briefly, these ILs are derived from RILs obtained from crosses between CB4856 and N2 (see [40], [45], [46] for details). This involved back-crossing RILs to N2, genotyping, and further back-crossing as appropriate, with lines finally genotyped at a total of 123 markers across the genome (20 each on chromosomes *I*, *II*, *III*, and *X*, 22 on *IV*, and 21 on *V*). Isolates were maintained using standard methods on NGM plates [47], with the OP50 strain of *Escherichia coli* as a food source. Unless otherwise stated, all assays were performed at 20°C and were initiated with fourth larval stage worms (L4s) grown from synchronized, arrested, L1s. Within each experiment, plates were blind coded and treatments were randomized such that the position within the incubator was not determined by genotype and that counts were done without knowledge of worm genotype. Plates that became contaminated with fungi and, for the population assays, on which worms failed to grow, were excluded from all analyses.

### Assays for dauer larvae formation in growing populations

Assays for population size and dauer larvae number at food exhaustion were performed as described by Green and Harvey [29] and Green et al [28], with assays undertaken using sloppy agar plates containing 4 g/l of agar [28]. For these assays, overnight cultures of OP50, grown in LB broth, were centrifuged and the supernatant discarded, with the bacterial pellet re-suspended in water at the required percentage w/v concentration and 100 µl of this suspension added to the plates. Plates were monitored daily until patch exhaustion, identifiable as the worms disperse from the exhausted area [29], [35], at which point the population size (total number of worms) and the number of dauer larvae was determined [29]. Dauer larvae were identified after incubation in 1% (w/v) solution of sodium dodecyl sulphate, a treatment that kills all non-dauer stages of *C. elegans* [17]. For these assays, data were analyzed by Kruskal Wallis test, with subsequent *post hoc* testing undertaken by pairwise Mann-Whitney U-tests or by ANOVA and *post hoc* testing by Fisher’s Least Significant Difference (LSD). To investigate the effect of variation at *nath-10* on dauer larvae numbers at food exhaustion, N2, JU1648, JU2041 and JU2047 were analyzed as described above in two replicate assays. Given the normality of residuals in these assays, these data could be analyzed by ANOVA, and Fisher’s LSD was used for *post hoc* testing.

For the assay of dauer larvae number a week after food exhaustion, plates with 100 µl of 20% w/v OP50 were monitored as described above to identify when food was exhausted. The number of dauer larvae formed at this time was then determined, as described above, for 8 plates each of N2 and of CB4856. Assay plates (n = 8 per line) were then returned to the incubator for seven days at which time the number of dauer larvae was determined. Note that the batch of OP50 used in this assay was grown using a different incubator than that used in other assays (above and [28]) and this allowed a much greater degree of aeration of the cultures. At comparable % w/v concentrations, bacteria grown under these conditions result in much lower levels of dauer larvae formation at food exhaustion than that observed with previous methods. For this assay, differences between ILs and N2 were investigated by Kruskal Wallis test, with *post hoc* testing undertaken by pairwise Mann-Whitney U-tests. IL versus IL comparisons used to define some of the growing population QTLs [28] were undertaken by Mann-Whitney U-tests with a Bonferroni adjustment of the *p* values used to correct for multiple testing.

### Assays for dauer larvae formation in response to defined amounts of pheromone

Dauer pheromone extract was prepared from N2 liquid culture media as previously described [27] except that culture supernatant was dried under reduced pressure at low temperature (40–60°C) in a rotary evaporator. A single batch of pheromone extract was used for all assays, which were carried out as previously described [23], [27] using 3.5 cm diameter plates with 2 ml of dauer larva formation assay agar, 25 µl of dauer pheromone extract and 20 µl of 1% w/v *E. coli* OP50 from overnight cultures that had been grown to saturation, had the media removed, and been resuspended in water. The genome-wide IL assay was undertaken in one experimental block, with 4 day old hermaphrodites transferred to assay plates, allowed to lay eggs, and then removed. Plates were then incubated at 25°C for two days at which point the numbers of dauer and non-dauer larvae on each plate were determined.

To test for CB4856 regions that affect dauer larvae development, two mapping approaches were taken. Firstly, the proportion of dauer and non-dauer larvae observed in each IL was compared to that observed in N2 by chi-square, using the proportions observed in N2 as the expected distribution. To correct for multiple testing the threshold for significance was determined by Bonferroni correction, giving a threshold of *p*≤6.58e-4 for each IL by N2 test. Comparisons between ILs were then made sequentially [48], with differences between IL pairs implying that chromosome segments not shared by the two ILs contain a QTL. For these IL by IL comparisons, a minimum spanning tree that connects the strains according to their pairwise similarity was constructed and the ILs were compared, by chi-square, using the proportions of dauer and non-dauer larvae in one IL as the expected distribution [28], [38]. Again, the threshold value for significance was determined by Bonferroni correction, giving a threshold of *p*≤8.92e-4 for each IL by IL test.

The retest of the ILs with introgressions on chromosome I and of JU1648 and JU2041 was undertaken as described above except that 40 µl of dauer pheromone extract was used per plate. For this assay, as the residuals were normally distributed, the arcsine square root transformed proportion data were analysed by ANOVA, with Fisher’s LSD used for *post hoc* testing.

## Supporting Information

Table S1 **Comparisons of dauer larvae formation in response to defined amounts of pheromone between introgression lines and N2.** Shown are the numbers of dauer larvae, the number of non-dauer larvae (L4s), the total number of worms, and the χ^2^ value from a comparison with dauer larvae formation in the N2 controls. Significant comparisons are noted and ‘effect of CB allele’ indicates if the CB4856 genotype at the QTL increases (P) or decreases (N) the number of dauer larvae in comparison to N2. The QTL ‘limits’ columns denote the maximum possible QTL regions defined by these comparisons.
(CSV)

Table S2 **Comparisons of dauer larvae formation in response to defined amounts of pheromone between introgression lines with overlapping regions of the CB4856 genome.** Shown are the ILs tested, the numbers of dauer larvae, the number of non-dauer larvae (L4s), the total number of worms, and the χ^2^ value from a comparison between the ILs. Significant comparisons are noted and ‘effect of CB allele’ indicates if the CB4856 genotype at the QTL increases (P) or decreases (N) the number of dauer larvae in comparison to N2. The QTL ‘limits’ columns denote the maximum possible QTL regions defined by these comparisons.
(CSV)

Table S3 **Locations and effect of QTLs detected for dauer larvae formation in response to defined amounts of pheromone.** ‘Line’ indicates the IL or the IL by IL test in which the QTL is detected, ‘effect’ indicates if the CB4856 genotype at the QTL increases (P) or decreases (N) the number of dauer larvae in comparison to N2. The ‘limits’ columns denote the possible QTL regions defined by these comparisons. ‘Overlap’ indicates QTLs that colocalize with QTLs affecting dauer larvae development in growing populations.
(CSV)

Table S4 **Comparisons of dauer larvae formation in response to defined amounts of pheromone between introgression lines with overlapping regions of the CB4856 genome on chromosome** ***I*** **.** Shown are the ILs tested, the numbers of dauer larvae, the number of non-dauer larvae (L4s), the total number of worms, and the results of post-hoc comparisons between IL by Fisher’s LSD. Significant comparisons are noted and ‘effect of CB allele’ indicates if the CB4856 genotype at the QTL increases (P) or decreases (N) the number of dauer larvae in comparison to N2. The QTL ‘limits’ columns denote the maximum possible QTL region defined by these comparisons.
(CSV)

File S1 **Raw data.** File A: Data for Table 1, retest of population size and dauer larvae number at food exhaustion of introgression lines at 20% w/v food. File B: Data for Figure 1, population size and dauer larvae number at food exhaustion of introgression lines at differing food concentrations. File C: Data for Figure 2, dauer larvae number a week after food exhaustion. File D: Data for Figure 3, population size and dauer larvae number at food exhaustion of *nath-10* lines. File E: Data for Figure 4, the likelihood of dauer larvae formation in the introgression lines. File F: Data for Figure 5, retest of likelihood of dauer larvae formation in chromosome I introgression lines and *nath-10* lines.
(ZIP)

## References

[1] Fisher RA (1918) The correlation between relatives on the supposition of Mendelian inheritance. Trans R Soc Edinb 52: 399–433.

[2] Gibson G (2010) Hints of hidden heritability in GWAS. Nat Genet 42: 558–560. 20581876 10.1038/ng0710-558

[3] Kammenga JE, Doroszuk A, Riksen JA, Hazendonk E, Spiridon L, et al (2007) A *Caenorhabditis elegans* wild type defies the temperature-size rule owing to a single nucleotide polymorphism in *tra-3* . PLoS Genet 3: e34. 17335351 10.1371/journal.pgen.0030034 PMC1808073

[4] Stern DL, Orgogozo V (2008) The loci of evolution: how predictable is genetic evolution? Evolution 62: 2155–2177. 18616572 10.1111/j.1558-5646.2008.00450.x PMC2613234

[5] Terpstra IR, Snoek LB, Keurentjes JJ, Peeters AJ, van den Ackerveken G (2010) Regulatory network identification by genetical genomics: signalling downstream of the *Arabidopsis* receptor-like kinase ERECTA. Plant Physiol 154: 1067–1078. 20833726 10.1104/pp.110.159996 PMC2971588

[6] Kammenga JE, Phillips PC, De Bono M, Doroszuk A (2008) Beyond induced mutants: using worms to study natural variation in genetic pathways. Trends Genet 24: 178–185. 18325626 10.1016/j.tig.2008.01.001

[7] Mackay TF, Stone EA, Ayroles JF (2009) The genetics of quantitative traits: challenges and prospects. Nat Rev Genet 10: 565–577. 19584810 10.1038/nrg2612

[8] Rockman MV (2012) The QTN program and the alleles that matter for evolution: all that’s gold does not glitter. Evolution 66: 1–17. 22220860 10.1111/j.1558-5646.2011.01486.x PMC3386609

[9] Schlichting CD, Pigliucci M (1998) Phenotypic Evolution: A Reaction Norm Perspective. Sunderland, MA: Sinauer Associates.

[10] Ghalambor CK, McKay JK, Carroll SP, Reznick DN (2007) Adaptive versus non-adaptive phenotypic plasticity and the potential for contemporary adaptation in new environments. Funct Ecol 21: 394–407.

[11] Gross MR (1996) Alternative reproductive strategies and tactics: diversity within sexes. Trends Ecol Evol 11: 92–98. 21237769 10.1016/0169-5347(96)81050-0

[12] Tomkins JL, Hazel W (2007) The status of the conditional evolutionarily stable strategy. Trends Ecol Evol 22: 522–528. 17919770 10.1016/j.tree.2007.09.002

[13] Moczek AP (2010) Phenotypic plasticity and diversity in insects. Philos Trans R Soc Lond B Biol Sci 365: 593–603. 20083635 10.1098/rstb.2009.0263 PMC2817146

[14] Pigliucci M (2005) Evolution of phenotypic plasticity: where are we going now? Trends Ecol Evol 20: 481–486. 16701424 10.1016/j.tree.2005.06.001

[15] Kaufmann K, Pajoro A, Angenent GC (2010) Regulation of transcription in plants: mechanisms controlling developmental switches. Nat Rev Genet 11: 830–842. 21063441 10.1038/nrg2885

[16] Pantin F, Simonneau T, Rolland G, Dauzat M, Muller B (2011) Control of leaf expansion: a developmental switch from metabolics to hydraulics. Plant Physiol 156: 803–815. 21474437 10.1104/pp.111.176289 PMC3177277

[17] Cassada RC, Russell RL (1975) The dauerlarva, a post-embryonic developmental variant of the nematode Caenorhabditis elegans. Dev Biol 46: 326–342. 1183723 10.1016/0012-1606(75)90109-8

[18] Kiontke KC, Félix M-A, Ailion M, Rockman MV, Braendle C, et al (2011) A phylogeny and molecular barcodes for *Caenorhabditis*, with numerous new species from rotting fruits. BMC Evol Biol 11: 339. 22103856 10.1186/1471-2148-11-339 PMC3277298

[19] Félix M-A, Duveau F (2012) Population dynamics and habitat sharing of natural populations of *Caenorhabditis elegans* and *C. briggsae.* . BMC Biol 10: 59. 22731941 10.1186/1741-7007-10-59 PMC3414772

[20] Petersen C, Dirksen P, Prahl S, Strathmann EA, Schulenburg H (2014) The prevalence of *Caenorhabditis elegans* across 1.5 years in selected North German locations: the importance of substrate type, abiotic parameters and *Caenorhabditis* competitors. BMC Ecol 14: 4. 24502455 10.1186/1472-6785-14-4 PMC3918102

[21] Hu PJ (2007) Dauer. In WormBook. Edited by The *C. elegans* Research Community. WormBook, http://www.wormbook.org.

[22] Viney ME, Gardner MP, Jackson JA (2003) Variation in *Caenorhabditis elegans* dauer larva formation. Dev Growth Differ 45: 389–396. 12950280 10.1046/j.1440-169x.2003.00703.x

[23] Harvey SC, Shorto A, Viney ME (2008) Quantitative genetic analysis of life-history traits of *Caenorhabditis elegans* in stressful environments. BMC Evol Biol 8: 15. 18211672 10.1186/1471-2148-8-15 PMC2267162

[24] Diaz SA, Brunet V, Lloyd-Jones GC, Spinner W, Wharam B, Viney M (2014) Diverse and potentially manipulative signalling with ascarosides in the model nematode *C. elegans* . BMC Evol Biol 14: 46. 24618411 10.1186/1471-2148-14-46 PMC4007702

[25] Mayer MG, Sommer RJ (2011) Natural variation in *Pristionchus pacificus* dauer formation reveals cross-preference rather than self-preference of nematode dauer pheromones. Proc R Soc Lond B Biol Sci 278: 2784–2790. 10.1098/rspb.2010.2760 PMC3145190 21307052

[26] Bose N, Meyer JM, Yim JJ, Mayer MG, Markov GV, et al (2014) Natural variation in dauer pheromone production and sensing supports intraspecific competition in nematodes. Curr Biol 24: 1536–1541. 24980503 10.1016/j.cub.2014.05.045 PMC4437242

[27] Golden JW, Riddle DL (1984) The *Caenorhabditis elegans* dauer larva: developmental effects of pheromone, food, and temperature. Dev Biol 102: 368–378. 6706004 10.1016/0012-1606(84)90201-x

[28] Green JWM, Snoek LB, Kammenga JE, Harvey SC (2013) Genetic mapping of variation in dauer larvae development in growing populations of *Caenorhabditis elegans* . Heredity 111: 306–313. 23715016 10.1038/hdy.2013.50 PMC3807260

[29] Green JWM, Harvey SC (2012) Development of *Caenorhabditis elegans* dauer larvae in growing populations. Nematology 14: 165–173.

[30] McGrath PT, Xu Y, Ailion M, Garrison JL, Butcher RA, Bargmann CI (2011) Parallel evolution of domesticated Caenorhabditis species targets pheromone receptor genes. Nature 477: 321–325. 21849976 10.1038/nature10378 PMC3257054

[31] Duveau F, Félix MA (2012) Role of pleiotropy in the evolution of a cryptic developmental variation in *Caenorhabditis elegans* . PLoS Biol 10: e1001230. 22235190 10.1371/journal.pbio.1001230 PMC3250502

[32] Stewart MK, Clark NL, Merrihew G, Galloway EM, Thomas JH (2005) High genetic diversity in the chemoreceptor superfamily of *Caenorhabditis elegans* . Genetics 169: 1985–1996. 15520260 10.1534/genetics.104.035329 PMC1449585

[33] Maydan JS, Flibotte S, Edgley ML, Lau J, Selzer RR, et al (2007) Efficient high-resolution deletion discovery in *Caenorhabditis elegans* by array comparative genomic hybridization. Genome Res 17: 337–347. 17267812 10.1101/gr.5690307 PMC1800925

[34] Maydan JS, Lorch A, Edgley ML, Flibotte S, Moerman DG (2010) Copy number variation in the genomes of twelve natural isolates of *Caenorhabditis elegans* . BMC Genomics 11: 62. 20100350 10.1186/1471-2164-11-62 PMC2822765

[35] Hodgkin J, Barnes T (1991) More is not better: brood size and population growth in a self-fertilizing nematode. Proc Roy Soc Lond B 246: 19–24. 10.1098/rspb.1991.0119 1684664

[36] Hodgkin J, Doniach T (1997) Natural variation and copulatory plug formation in *Caenorhabditis elegans* . Genetics 146: 149–164. 9136008 10.1093/genetics/146.1.149 PMC1207933

[37] Gutteling EW, Riksen JA, Bakker J, Kammenga JE (2007) Mapping phenotypic plasticity and genotype-environment interactions affecting life-history traits in *Caenorhabditis elegans* . Heredity 98: 28–37. 16955112 10.1038/sj.hdy.6800894

[38] Snoek LB, Orbidans HE, Stastna JJ, Aartse A, Rodriguez M, et al (2014) Widespread genomic incompatibilities in *Caenorhabditis elegans* . G3 (Bethesda) 4: 1813–1823. 25128438 10.1534/g3.114.013151 PMC4199689

[39] Harvey SC (2009) Non-dauer larval dispersal in *Caenorhabditis elegans* . J Exp Zool B Mol Dev Evol 312: 224–230. 10.1002/jez.b.21287 19288538

[40] Rodriguez M, Snoek LB, Riksen JA, Bevers RP, Kammenga JE (2012) Genetic variation for stress-response hormesis in *C. elegans* lifespan. Exp Gerontol 47: 581–487. 22613270 10.1016/j.exger.2012.05.005

[41] Andersen EC, Gerke JP, Shapiro JA, Crissman JR, Ghosh R, et al (2012) Chromosome-scale selective sweeps shape *Caenorhabditis elegans* genomic diversity. Nat Genet 44: 285–290. 22286215 10.1038/ng.1050 PMC3365839

[42] Glater EE, Rockman MV, Bargmann CI (2014) Multigenic natural variation underlies *Caenorhabditis elegans* olfactory preference for the bacterial pathogen *Serratia marcescens* . G3 (Bethesda) 4: 265–276. 24347628 10.1534/g3.113.008649 PMC3931561

[43] Rajon E, Plotkin JB (2013) The evolution of genetic architectures underlying quantitative traits. Proc R Soc B 280: 20131552. 10.1098/rspb.2013.1552 PMC3768309 23986107

[44] Doroszuk A, Snoek LB, Fradin E, Riksen J, Kammenga J (2009) A genome-wide library of CB4856/N2 introgression lines of *Caenorhabditis elegans* . Nucleic Acids Res 37: e110. 19542186 10.1093/nar/gkp528 PMC2760803

[45] Li Y, Alvarez OA, Gutteling EW, Tijsterman M, Fu J, et al (2006) Mapping determinants of gene expression plasticity by genetical genomics in *C. elegans* . PLoS Genet 2: e222. 17196041 10.1371/journal.pgen.0020222 PMC1756913

[46] Li Y, Breitling R, Snoek LB, van der Velde KJ, Swertz MA, et al (2010) Global genetic robustness of the alternative splicing machinery in *Caenorhabditis elegans* . Genetics 186: 405–10. 20610403 10.1534/genetics.110.119677 PMC2940304

[47] Stiernagle T (2006) Maintenance of *C. elegans* In WormBook. Edited by The *C. elegans* Research Community. WormBook, http://www.wormbook.org.

[48] Shao H, Sinasac DS, Burrage LC, Hodges CA, Supelak PJ, et al (2010) Analyzing complex traits with congenic strains. Mamm Genome 21: 276–286. 20524000 10.1007/s00335-010-9267-5 PMC3805105

